# Diagnostic value of methane-hydrogen breath test combined with intestinal flora testing in screening for colorectal polyps

**DOI:** 10.3389/fmed.2025.1530558

**Published:** 2025-05-30

**Authors:** Wei Wang, Hui Li, Zhihui Yan, Rui Li, Yan Zheng, Xiaohui Wang, Lihong Cui

**Affiliations:** ^1^Chinese PLA Medical School, Beijing, China; ^2^Senior Department of Gastroenterology, Chinese PLA General Hospital, Beijing, China

**Keywords:** methane-hydrogen breath test, colorectal polyps, intestinal barrier function, subject working curve preamble, screening

## Abstract

**Purpose:**

Diagnostic value of Methane and Hydrogen Breath Test (MHBT) combined with intestinal flora detection in colorectal polyp screening.

**Methods:**

Retrospective inclusion of 196 patients who had completed MHBT with colonoscopy during the same period of time in the General Hospital of the People’s Liberation Army (PLA), China, from January 2022 to December 2023, were divided into a control group (no polyps) and an observation group (with polyps, *n* = 109). A total of 196 patients who were examined were divided into control group (without polyps, *n* = 109) and observation group (with polyps, *n* = 87). Baseline data, intestinal barrier function (DAO, D-lac, LPS), tumor markers (AFP, CA19-9, CA-125, CEA), MHBT positivity rate and relative abundance of intestinal flora were analyzed in the two groups, and diagnostic efficacy was assessed by ROC curve.

**Results:**

The proportion of males (64.94 vs. 49.54%) and age (61.85 ± 12.38 vs. 52.47 ± 13.57 years) in the observation group were significantly higher than those in the control group (*P* < 0.05). After multifactorial correction, CH_4_ (OR = 2.32, *P* = 0.019) and H_2_ positivity (OR = 2.14, *P* = 0.027) remained significantly higher in the observation group. In the observation group, *Bifidobacterium* spp. (Bb, –15.91 ± 2.86 vs. –16.65 ± 2.13 in the control group, *t* = 2.075, *P* = 0.039), *Lactobacillus* spp. (Lb, –12.58 ± 3.67 vs. –15.87 ± 2.70, *t* = 6.988, *P* < 0.001), enterotoxin-producing Enterotoxin fragile mimics (ETBF, –6.02 ± 2.17 vs. 6.02 ± 2.17 vs. –6.69 ± 2.23, *t* = 2.122, *P* = 0.035), Clostridium nucleatum (Fn, –18.73 ± 2.88 vs. –21.28 ± 3.07, *t* = 5.984, *P* < 0.001) and anaerobic Streptococcus pepticus (Panaerobius, –16.23 ± 1.98 vs. – 20.30 ± 2.43, *t* = –2.916, *P* < 0.001) were significantly higher than the relative quantitative values of the control group. ROC analysis showed that the AUC for diagnosing colorectal polyps with CH_4_, H_2_ and the combined assay (CH_4_+H_2_) were 0.725, 0.640, and 0.768, respectively; and after combining the intestinal flora (Bb, Lb, Fn, etc.) AUC was elevated to 0.831, with a sensitivity of 79.27% and a specificity of 82.90%.

**Conclusion:**

HBT provides a non-invasive strategy for colorectal polyp screening by capturing CH_4_/H_2_ metabolic gases with intestinal flora characteristics. Combining flora markers significantly improves diagnostic efficacy, suggesting its translational potential in optimizing screening pathways. Future exploration of the mechanisms of flora-metabolic gas dynamic interactions and individualized threshold setting is warranted.

## 1 Introduction

In recent years, with the improvement of China’s economic level, culture and material life have undergone great changes, coupled with the change of dietary structure and the influence of bad habits, digestive system diseases are on the rise year by year, and the incidence rate of digestive system tumors is also increasing year by year. Colorectal cancer is a common malignant tumor that ranks fourth among malignant tumors of the digestive system, and its mortality rate ranks among the top three in the world, with more than 900,000 people dying of colorectal cancer every year (1, 2). It is expected that by 2023, the number of colorectal cancer cases worldwide could increase by about 60% compared to the current level, with 2.2 million new cases and 1.1 million deaths due to colorectal cancer (3). There is evidence of a strong association between colorectal cancer and colorectal polyps. Colorectal polyps are prominent growths on the mucosal surface of the intestinal tract, the incidence of which increases with age and is particularly prevalent in people over the age of 40 Although most polyps are benign (4), adenomatous polyps have been shown to be an early stage of colon cancer, with the cancer rate of these polyps ranging from 0.2 to 5.0% (5). Most colorectal cancers are the gradual evolution of benign polyps, a process that involves genetic, histologic, morphologic, and intestinal microbiome changes and usually takes more than 10 years (6, 7). Emphasizing the early diagnosis of colorectal polyps, especially the prevention, early diagnosis and treatment of adenomatous polyps, is important for improving the overall prognosis of colorectal cancer patients. Currently including fecal occult blood test (FOBT), flexible sigmoidoscopy, and colonoscopy are being used for colorectal cancer screening with different advantages and disadvantages. FOBT and sigmoidoscopy screening reduced the mortality rate of colorectal cancer by 16 and 30%, respectively (6); observation of the lesion, biopsy and diagnosis is preferred to colonoscopy (7), but the modality is invasive diagnosis, expensive, limited frequency of examination, and low patient compliance. There is an urgent need to explore an accurate, easy to perform, non-invasive and highly reproducible screening tool for colorectal cancer and colorectal polyps. Gut microbiota, as an important player in intestinal health, has gradually gained attention for its association with gastrointestinal diseases, especially colorectal cancer and its precancerous lesions. In recent years, it has been found that dysregulation of the composition of the intestinal flora is closely associated with the development of adenomatous polyps. MHBT is a reliable way to measure GI bacteria (8), and although jejunal aspirate culture is still regarded as the gold standard for the diagnosis of small intestinal bacterial overgrowth (SIBO), the test is complicated and invasive and MHBT has been, by virtue of its safety, cost-effectiveness, and simplicity of operation, been widely used for clinical screening of SIBO. Although some studies have questioned its accuracy and specificity, especially its consistency across different substrates and populations, it still has some clinical reference value as a non-invasive tool. Recent studies have shown (9–11) an association between intestinal microorganisms and the development of adenomatous polyps and direct bowel cancer. In this study, we combined the molecular detection techniques of intestinal flora to analyze the relative abundance of *Bifidobacterium* spp. (Bb), *Lactobacillus* spp. (Lb), Enterotoxin-producing Bacteroidetes fragilis (ETBF), Clostridium nucleatum (Fn), and anaerobic Streptococcus alginolyticus (Panaerobius) by real-time fluorescence quantitative PCR (qPCR), and then to explore the correlation between the two species and the development of colorectal polyps, and then evaluate the clinical value of MHBT combined with intestinal flora testing for colorectal polyp screening was evaluated. The results of the study aim to provide a more comprehensive and precise non-invasive strategy for colorectal polyp screening, optimize the existing pathway for colorectal polyp screening, and provide a new evidence-based basis for early detection and prevention of colorectal cancer.

## 2 General information, methods

### 2.1 General information

A retrospective study was conducted on 196 MHBT patients admitted to the Department of Gastroenterology of the General Hospital of the Chinese People’s Liberation Army (PLA) from January 2022 to December 2023, grouped according to the presence or absence of colorectal polyps, with those who had a normal colorectal mucosa included in the control group (*n* = 109), and those who developed colorectal polyps included in the observation group (*n* = 87). Inclusion criteria for the control group: age > 18 years; no colorectal polyps or other intestinal lesion s were found by colonoscopy; inclusion criteria for the observation group: age > 18 years; colorectal polyps observed by colonoscopy and confirmed by pathological biopsy. Exclusion criteria: those with acute intestinal infectious disease s; antibiotic users in the last 2 weeks; (capsule) endoscopy user in the last 2 weeks; those who could not tolerate colonoscopy; those with combined serious dysfunction of other organs; and those aged < 18 years.

### 2.2 Research design

This was a single-center retrospective cohort study that included 196 consecutive patients who completed HMBT and colonoscopy between January 2022 and December 2023 during the same period. To clarify the timing of testing: all patients were prioritized for completion of HMBT testing (median interval 3 d, IQR 2∼ 5 d); colonoscopy was completed within 7 d of the breath test; endoscopy operators were blinded to the results of the breath test, and laboratory technicians were uninformed about the results of the colonoscopy.

### 2.3 Subject preparation

Implementation of a standardized pretreatment regimen: dietary control: low fermentable oligo-, di-, mono-, and polyol (FODMAP) diet for 24 h prior to testing; medication restriction: discontinuation of proton pump inhibitors (≥ 7 d), antibiotics/probiotics (≥ 4 weeks), and laxatives (≥ 72 h); and fasting requirements: 12 h of fasting prior to testing, prohibition of smoking and alcohol consumption

### 2.4 Detection process

MHBT: Detection was performed using a Quintron SC gas chromatograph (Quintron, United States): baseline value collection: three breath samples were collected during morning fasting; substrate stimulation: oral administration of 20 g of lactulose solution (INALCO, batch no. 17573) Dynamic monitoring: breath samples were collected every 15 min for 180 min; diagnostic criteria [refer to North American Consensus (12)]: H_2_ positivity: H*2* rise ≥ 20 ppm in 90 min, CH_4_ positivity: CH*4* ≥ 10 ppm at any time point.

Colonoscopy: completed by an endoscopist with more than 5 years of experience using an Olympus CF-HQ190 enteroscope: quality of bowel preparation: assessed using the Boston Bowel Preparedness Scale (BBPS ≥ 6 was considered satisfactory); polyp characterization record: site, size, and morphologic features were recorded according to Parisian typing; pathological diagnosis: diagnostic results were blindly reviewed by two independent pathologists.

### 2.5 Fecal microbial genome extraction and qPCR primer preparation

Extraction of genomic DNA from fecal samples and determination of its concentration and purity: The extraction of microbial genomic DNA from fecal samples was carried out in a biosafety cabinet using the TIANamp Stool DNA extraction kit provided by Tiangen Biochemistry Technology (Beijing) Co. The concentration of the resulting DNA solution and its purity were detected and evaluated by a NanoDrop 2000C UV-Vis spectrophotometer manufactured by Thermo Electron, United States.

Primer design and synthesis: The primer sequences of the target and internal reference genes were obtained from the relevant research data, and analyzed by the National Center for Biotechnology Information (NCBI) Nucleic Acid Sequence Alignment Tool (BLAST) for specificity comparison. The primer synthesis was undertaken by KeKing Biotechnology Co., Ltd. and after specificity verification, the primer pairs that were most suitable for subsequent experiments were screened out, and the specific information is shown in Table 1.

**TABLE 1:** Primer sequences.

Primer name	Upstream primers (5′–3′)	Downstream primers (5′–3′)
Uni-bacteria	–CGGCAACGAGCGCAACCC–	–CCATTGTAGCACGTGTGTAGCC–
Lb	–AGCAGTAGGGAATCTTCCA–	–CACCGCTACACATGGAG–
Bb	–CTTACTTCGCCTTCTTTGCTCCTTAC–	–AGAAGTCCAAGACTTTGGCCCTGA–
Fn	–CTTAGGAATGAGACAGAGATG–	–TGATGGTAACATACGAAAGG–
ETBF	–TAGAGTTAGACCCTACCCGT–	–CGGCTACAGAACAGAACC–
Panaerobius	–CTGGTGGATAGGAGGCAAAG–	–CCACAATATTGGCATTTGGA–

Uni-bacteria are internal reference genes; Lb is *Lactobacillus* spp; Bb is *Bifidobacterium* spp; Fn is Clostridium nucleatum; ETBF is enterotoxin-producing Enterotoxin fragile mimic; Panaerobius is anaerobic digestive streptococcus.

### 2.6 Evaluation indicators

Diagnostic criteria for SIBO: methane-hydrogen breath test judgment criteria according to the methane-hydrogen breath test with reference to the Rome Consensus, the North American Consensus (12, 13): hydrogen value ≥ 20 ppm within 90 min is positive for hydrogen, then should be considered as the presence of SIBO; methane value ≥ 10 ppm within 90 min is methane-positive, and is considered to be the presence of SIBO. Any of the above criteria can be diagnosed as SIBO.

Intestinal barrier function (13): Normal reference values for diamine oxidase (D AO), D-lactic acid (D-LAC), and bacterial endotoxin (LPS) are ≤ 10 U/L, ≤ 15 mg/L, and ≤ 20 U/L, respectively; any one of these indexes exceeding the norm al reference value is considered to be the presence of impaired intestinal barrier function.

Tumor four: using enzyme-linked immunosorbent assay (ELISA) to detect alpha-fetoprotein (AFP), in accordance with the normal reference value set by the Department of Laboratory Medicine of the hospital: ≤ 9 ng/mL, glycan antigen 125 (CA-125), glycan antigen 19-9 (CA19-9), carcinoembryonic antigen (C EA) detection principle for the antigen-antibody reaction, in accordance with the Department of Laboratory Medicine of the hospital, respectively, set the nor mal reference value as follows ≤ 35 U/mL, ≤ 25 U/mL, ≤ 5 ng/mL.

### 2.7 Statistical methods

The STROBE observational study reporting specifications were followed: ROC curve analysis using MedCalc 20.0 software (DeLong method for comparing AUC differences); multifactorial logistic regression to correct for confounders such as age and gender (variables with *P* < 0.1 for univariate analysis were included); and diagnostic performance assessment: calculation of the sensitivity, specificity, and Youden’s Index optimal cutoff values; *P*< 0.05 was considered a statistically significant difference.

## 3 Results

### 3.1 One-way analysis of baseline data for both groups

The results of the study showed that there was no significant difference in the incidence of intestinal barrier function, four tumor items and constipation between the two groups (*P* > 0.05); the proportion of male patients in the observation group was higher than that in the control group, and the age of patients was greater than that in the control group (*P* < 0.05) (see Table 2).

**TABLE 2:** One-way analysis of baseline data in the two groups [*n*, (%); (_x_ ± s)].

Sports event		Control group (*n* = 109)	Observation group (*n* = 87)	t/χ^2^	*P*
Distinguishing between the sexes	Male	54 (49.54)	56 (64.94)	4.574	0.033
	Daughter	56 (51.38)	31 (35.06)		
(A person’s) age		52.47 ± 13.57	61.85 ± 12.38	4.997	<0.001
Impaired intestinal barrier function	Be	59 (54.13)	50 (57.47)	0.218	0.641
	Clogged	50 (45.87)	37 (42.53)		
DAO		5.78 ± 7.15	5.13 ± 7.70	–0.611	0.542
D-lac		15.53 ± 8.84	15.96 ± 8.78	0.339	0.735
LPS		8.53 ± 8.79	10.48 ± 10.25	1.433	0.154
Tumor IV	AFP (ng/mL)	3.58 ± 1.87	4.08 ± 2.13	1.748	0.082
	CA-125 (μ/mL)	24.22 ± 8.49	25.28 ± 7.43	0.917	0.360
	CA19-9 (μ/mL)	15.61 ± 4.18	16.20 ± 5.91	0.817	0.415
	CEA (ng/mL)	3.04 ± 1.92	3.44 ± 1.87	1.466	0.144
Insomnia	Be	95 (87.16)	68 (78.16)	2.782	0.095
	Clogged	14 (12.84)	19 (21.84)		

### 3.2 Comparison of CH_4_, H_2_, and CH_4_+H_2_ positivity in MHBT between the two groups

The results of the study showed that the incidence of CH_4_, H_2_ and CH_4_+H_2_ positivity was higher in the observation group than in the control group (*P* < 0.05) (see Table 3).

**TABLE 3:** Comparison of CH_4_, H_2_ and CH_4_+H2positivity rates in MHBT between the two groups [*n*,(%)].

Sports event		Control group (*n* = 109)	Observation group (*n* = 87)	χ^2^	*P*
CH_4_	+	64 (58.72)	63 (72.41)	3.960	0.047
	–	45 (41.28)	24 (27.59)		
H_2_	+	44 (40.37)	52 (59.77)	7.252	0.007
	–	65 (59.63)	35 (40.23)		
CH_4_+H_2_	+	26 (23.85)	40 (45.98)	10.549	0.001
	–	83 (76.15)	47 (54.02)		

### 3.3 Comparison of CH_4_ and H_2_ positivity in exhaled breath of control age and sex subgroups

**FIGURE 1:**
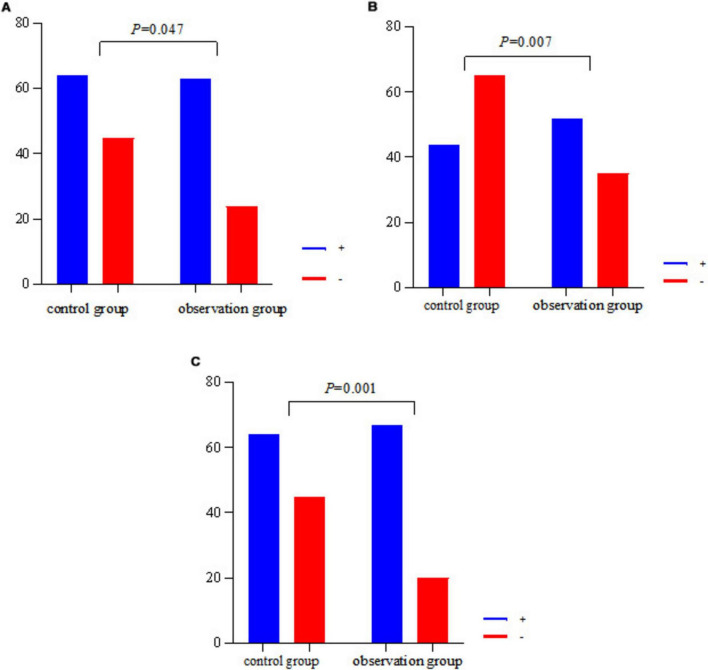
Histogram of CH_4,_ H_2,_ CH_4_+H_2_ positivity in two groups. **(A)** The CH_4_ positivity rate for both groups. **(B)** Plots the H_2_ positivity rate for both groups. **(C)** The CH_4_+H_2_ positivity rate for both groups.

Since there were significant differences in age and gender between the observation group and the control group, in order to control their effects on the results of the breath test, multifactorial logistic regression analyses were performed with CH_4_ and H_2_ positivity as the dependent variables, and group, age, and gender as the independent variables, respectively. The results showed that after controlling for the effects of age and gender, group remained a significant influence on CH_4_ and H_2_ positivity, and the rates of CH_4_ and H_2_ positivity in the observation group were significantly higher than those in the control group (*P* < 0.05) (see Tables 4, 5 and Figure 1).

**TABLE 4:** Multifactorial logistic regression analysis of the effect of group, sex, and age on positivity of breath CH*4*.

Variant	β	OR (95% CI)	*P*
Group (observation vs. control)	0.842	2.32 (1.15-4.67)	0.019
Age (years)	0.012	1.01 (0.98-1.03)	0.481
Gender (male vs. female)	0.293	1.34 (0.65-2.75)	0.428

**TABLE 5:** Logistic regression analysis of the effect of age, gender and group on exhaled H*2* positivity.

Variant	β	OR (95% CI)	*P*
Group (observation vs. control)	0.761	2.14 (1.09-4.22)	0.027
Age (years)	–0.007	0.99 (0.97-1.02)	0.593
Gender (male vs. female)	0.215	1.24 (0.61-2.51)	0.554

### 3.4 Comparison of relative quantification of intestinal flora between the two groups

**FIGURE 2:**
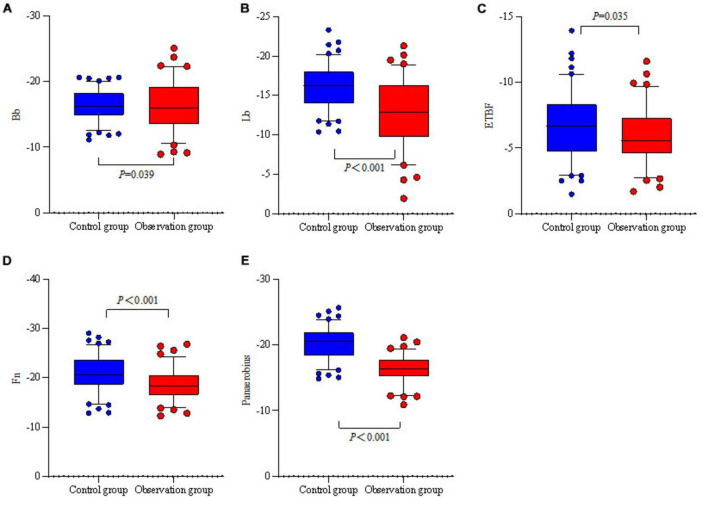
Relative quantitative box-and-whisker plots of the intestinal flora of the two groups. **(A)** The relative quantification of two groups of Bb. **(B)** The relative quantification of two groups of Lb. **(C)** The relative quantification of two groups of ETBF. **(D)** The relative quantification of two groups of Fn. **(E)** The relative quantification of two groups of Panaerobius.

Bb, Lb, ETBF, Fn, and Panaerobius were higher in the observation group than in the control group (*P* < 0.05) (see Table 6 and Figure 2).

**TABLE 6:** Comparison of relative quantification of intestinal flora between the two groups (–△ Ct).

Groups	Number of examples	Bb	Lb	ETBF	Fn	Panaerobius
Control subjects	109	–16.65 ± 2.13	–15.87 ± 2.70	–6.69 ± 2.23	–21.28 ± 3.07	–20.30 ± 2.43
Observation Group	87	–15.91 ± 2.86	–12.58 ± 3.67	–6.02 ± 2.17	–18.73 ± 2.88	–16.23 ± 1.98
*t*		2.075	6.988	2.122	5.984	–2.916
*P*		0.039	<0.001	0.035	<0.001	<0.001

### 3.5 Diagnostic efficacy analysis of CH_4_, H_2_, and CH_4_+H_2_ on colorectal polyps

**FIGURE 3:**
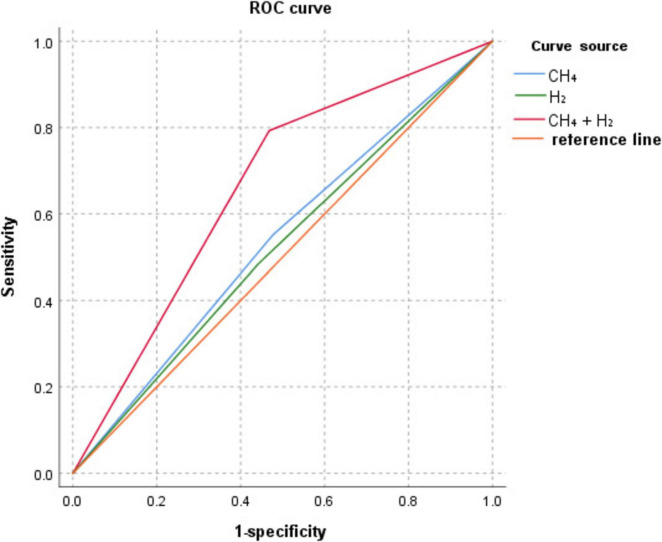
ROC curves of CH_4_, H_2_, and CH_4_+H_2_ for the diagnosis of colorectal polyps.

ROC curve analysis showed that the AUC for CH_4_, H_2_, and CH_4_+H_2_ diagnosis colorectal polyps were: 0.537, 0.521, and 0.663, as shown in Table 7 and Figure 3.

**TABLE 7:** Diagnostic efficacy analysis of CH_4_, H_2_ and CH_4_+H2on colorectal polyps.

Sports event	AUC	*P*	95% CI	Sensitivity	Idiosyncrasy	Jordon index (math.)
CH_4_	0.537	0.300	0.465∼0.609	55.17	52.29	0.464
H_2_	0.521	0.556	0.449∼0.593	48.28	55.96	0.042
CH_4_+H_2_	0.663	<0.001	0.592∼0.728	79.31	53.21	0.325

### 3.6 Diagnostic efficacy analysis of CH_4_+H_2_ combined with relative quantification of intestinal flora for colorectal polyps

**FIGURE 4:**
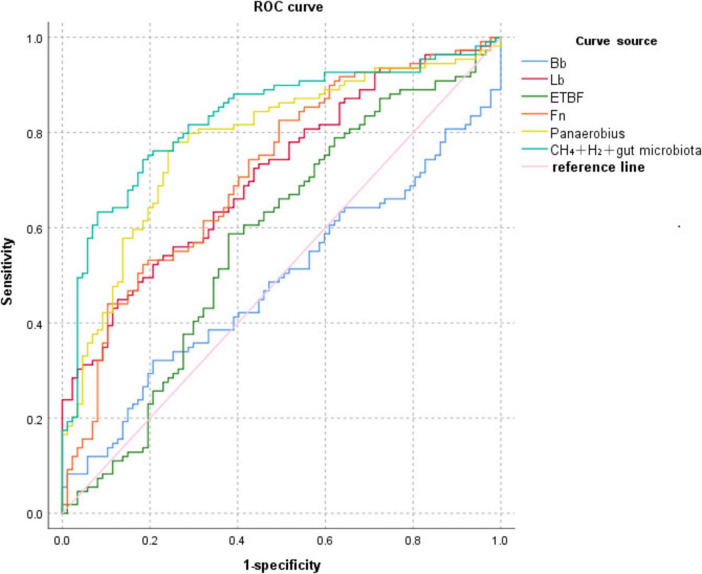
ROC curve of CH_4_+H_2_ combined with relative quantification of intestinal flora for the diagnosis of colorectal polyps.

ROC curve analysis showed that Bb, Lb, ETBF, Fn, Panaerobius, and CH_4_+ H_(2)_+ intestinal flora diagnosed colorectal polyps with AUC: 0.487, 0.713, 0.578, 0.717, 0.783, 0.831, as shown in Table 8 and Figure 4.

**TABLE 8:** Diagnostic efficacy analysis of CH_4_+H_2_ combined with relative quantification of intestinal flora for colorectal polyps.

Sports event	AUC	*P*	95% CI	Sensitivity	Idiosyncrasy	Jordon index (math.)	Truncation value
Bb	0.487	0.750	0.406∼0.568	63.41	50.32	0.562	≥-16.08
Lb	0.713	0.000	0.643∼0.784	73.25	64.52	0.123	≥-12.09
ETBF	0.578	0.060	0.496∼0.660	46.83	63.23	0.100	≥-7.17
Fn	0.717	0.000	0.645∼0.789	78.29	68.52	0.128	≥-19.22
Panaerobius	0.783	0.000	0.718∼0.849	92.68	60.00	0.127	≥-15.94
CH*4*+H*2*+ gutmicrobiota	0.831	0.000	0.773∼0.890	79.27	82.90	0.236	–

## 4 Discussion

Although there was no significant difference (*P* > 0.05) between the observation and control groups in systemic barrier function indicators such as DAO, D-lac, and LPS, the significantly higher CH_4_+H_2_ positivity may involve the following mechanisms: local barrier damage is prioritized over systemic alterations: colorectal polyps may mainly cause down-regulation of localized tight junction proteins in the mucosa (e.g., ZO-1) rather than widespread leaky gut. Animal experiments showed that claudin-3 expression was reduced by 50% within 5 mm of polyp margins, but serum DAO remained normal (14), consistent with the present findings. Selective diffusion of flora metabolites: CH_4_ and H_2_, as small molecule gases, can diffuse directly into the bloodstream through the locally damaged mucosa, whereas large molecule barrier markers (e.g., LPS) need to be extensively leaked to be significantly elevated. This may explain why there was a significant difference in breath positivity (CH_4_+H_2_ positivity 67/87 in the observation group vs. 64/109 in the control group, *P* = 0.007), but only a trend of elevation in LPS (10.48 vs. 8.53, *P* = 0.154). AFP, CA19-9, CA-125, and CEA did not differ between the two groups (*P* > 0.05). Combined with the pathology, it was confirmed that all of the observation groups were benign polyps, and the following mechanisms could explain the above results: MHBT positivity precedes tumor marker changes: dysbiosis may be an early event in colorectal carcinogenesis, while traditional tumor markers tend to be elevated after malignant transformation. Polimeno et al. (15) showed that p-STAT3 and ALR were found to be significantly up-regulated in the tissues of G1-stage CRC, while PIAS3 suppressed the expression of p-STAT3 and ALR. While PIAS3 suppressed expression, supporting the idea that colony-initiated signaling pathways are activated early in carcinogenesis, suggesting that colony dysregulation and its signaling pathways precede changes in conventional tumor markers. This study suggests that MHBT may serve as an early warning indicator of the malignant tendency of polyps, earlier than the abnormalities of markers such as CEA. The independent pathways of flora metabolism and tumor markers: MHBT reflects the functional status of the flora, while CEA and other markers are mainly associated with epithelial cell malignancy, and their synergistic effects have not yet formed in the polyp stage, so long-term follow-up is needed to verify their dynamic association. Although systemic barrier markers did not show any difference, the following mechanisms may link the local changes with the breath test results: regional immune microenvironment regulation: localized Th17/Treg imbalance in polyps may promote the proliferation of methanogenic bacteria, while IL-22 restricts the systemic diffusion of bacterial products through the up-regulation of MUC2, resulting in the phenotype of “localized bacterial flora activity—normal systemic barrier” (Figure 2) “Phenotype (16). Self-sustaining effects of bacterial metabolism (17): methane slows peristalsis by inhibiting smooth muscle contraction, prolonging the contact time between hydrogen-producing bacteria and substrate, resulting in a “low peristalsis-high gas production” cycle, a process that may be independent of classical barrier markers.

Although univariate analysis showed that the observation group was older (61.85 vs. 52.47 years) and had a higher proportion of males (64.94% vs. 49.54%), there was no significant effect of age (CH_4_: *P* = 0.481; H_2_: *P* = 0.593) and gender (CH_4_: P = 0.428; H_2_: *P* = 0.554) in the multivariate model. This may be explained by the fact that age is not a driver of breath positivity: despite the fact that aging is accompanied by a decline in the diversity of the intestinal flora, the present study confirms, by statistical correction, that the increased rate of breath positivity in patients with polyps is mainly attributable to the disease state and not to age-related changes in the physiological flora. The clinical significance of gender differences is limited: the high prevalence of males in colorectal polyps may stem from differences in hormone levels (e.g., androgens promote cell proliferation) or lifestyle habits (e.g., smoking/alcohol consumption) (18), but these factors did not directly interfere with flora gas-producing functions, and thus gender was not statistically significant in the multifactorial model. Multifactorial logistic regression analysis showed that after controlling for age and gender confounders, group (observation vs. control) remained an independent risk for positive CH_4_ (OR = 2.32, 95%CI = 1.15-4.67, *P* = 0.019) and H_2_ (OR = 2.14, 95%CI = 1.09-4.22, *P* = 0.027) Factors. This result suggests that the elevated breath test positivity in patients with colorectal polyps is not due to age or gender differences, but is closely related to pathophysiologic changes induced by the polyps themselves. Possible mechanisms include: colony-specific enrichment: the polyp microenvironment (e.g., local hypoxia, altered mucus secretion) may selectively promote the colonization of methanogenic bacteria (e.g., *Methanobrevibacter*) and hydrogen-producing bacteria (e.g., *Bacteroides*) (19). Girardi et al. (20) demonstrated in an animal model that adenomatous polyps The abundance of Pseudomonas aeruginosa (*Methanobrevibacter*) in tissues was 5∼ 8-fold higher than in normal mucosa. Metabolite feedback regulation: H_2_ produced by polyp-associated flora may promote the proliferation of pathogenic bacteria (e.g., *Fusobacterium nucleatum*) via the hydrogenase pathway, whereas CH_4_ may inhibit the contraction of intestinal smooth muscle and delay the transport of contents, forming a positive feedback loop (e.g., “dysbiosis-increased gas production”). Positive feedback loop (21).

The significant enrichment of Clostridium perfringens and Streptococcus anaerobius digestans in the observation group (*P* < 0.001) may have driven the increase in CH_4_/H_2_ positivity through the following mechanism: metabolic substrate competition: as a hydrogenotroph, Fn can consume H_2_ to produce H2S via hydrogenase, while methanogenic bacteria such as Panaerobius utilize H_2_/CO_2_ to synthesize CH_4_ (21), which together resulted in a simultaneous increase in H2 positivity (59.77% vs. 40.37%, *P* = 0.007) and CH_4_ positivity (72.41 vs. 58.72%, *P* = 0.047) in the observed group. Local barrier damage: although there was no difference in systemic barrier indicators (DAO, LPS), Fn-secreted FadA protein disrupted colonic epithelial tight junctions (22) and facilitated diffusion of small-molecule gases (CH_4_/H_2)_ into the bloodstream, whereas large-molecule toxins (e.g., LPS), which were blocked by intact mucosa, were not significantly elevated (10.48 vs. 8.53, *P* = 0.154). Notably, conventional probiotics such as *Bifidobacterium* spp. and *Lactobacillus* spp. proliferated abnormally in the observed group, possibly related to the altered pH of the polyp microenvironment or disruption of the mucus layer (23). Whether the overcolonization of such “probiotics” has pro-inflammatory or carcinogenic potential needs to be further verified by functional experiments.

Although the AUC of single CH_4_ assay was only 0.537 (*P* = 0.300), its combination with H_2_ elevated the AUC to 0.663 (*P* < 0.001), with a sensitivity of 79.31%, suggesting the following optimization logic: functional complementarity: CH_4_ labels the chronic dysbiosis of methanogens (specificity 52.29%), while H_2_ reflects the parthenogenicity of the acute metabolic activation of anaerobic bacteria (sensitivity 48.28%), and the combination of the two can cover a broader range of flora functional states. Enhanced specificity by flora markers: the introduction of genera strongly associated with polyps, such as Fn (AUC = 0.717) and Panaerobius (AUC = 0.783), significantly increased the AUC of the combined model to 0.831 (sensitivity 79.27%, specificity 82.90%). For example, Panaerobius ≥ –15.94 identified patients with high-risk polyps (sensitivity 92.68%), compensating for the high false-positive rate of a single breath test.

This study suggests that MHBT combined with flora analysis can be used as a stratified screening tool before colonoscopy: initial screening of high-risk groups: those with positive CH_4_+H_2_ (AUC = 0.663) or abnormal Panaerobius abundance (AUC = 0.783) are prioritized to undergo colonoscopy, reducing overuse of invasive tests. Value of dynamic monitoring: longitudinal monitoring of CH_4_/H_2_ changes may warn of malignant transformation of polyps, but needs to be validated in follow-up studies. Limitations include: threshold controversy: current CH_4_ diagnostic cutoff ( ≥ 10 ppm) has low specificity (52.29%) and needs to be recalibrated based on colony characteristics (e.g., specificity increased to 75% for CH_4_ ≥ 15 ppm). Undifferentiated polyp subtypes: possible differences in colony-breath characteristics between adenomatous and hyperplastic polyps, requiring subsequent stratified analysis.

## 5 Conclusion

In conclusion, MHBT can reflect the possible dysfunction of flora in patients with colorectal polyps by detecting the status of intestinal gas production and has certain screening value. The combined test of CH_4_ and H_2_ is superior to the single index in diagnostic efficacy, and has certain screening potential. Although its diagnostic specificity is still limited and it cannot yet replace colonoscopy, it can be used as a non-invasive aid for early detection of colorectal polyps in specific populations. Follow-up studies may further explore individualized threshold optimization, dynamic detection modes and their combined application with other biomarkers to improve screening accuracy and clinical utility.

## Data Availability

The original contributions presented in this study are included in this article/supplementary material, further inquiries can be directed to the corresponding authors.
